# Descriptive study of mental health care users 12 months pre- and post-COVID-19 lockdown

**DOI:** 10.4102/sajpsychiatry.v30i0.2301

**Published:** 2024-10-18

**Authors:** Noluthando A. Hlongwane, Karishma Lowton

**Affiliations:** 1 Department of Psychiatry, Faculty of Health Sciences, University of the Witwatersrand, Johannesburg, South Africa

**Keywords:** COVID-19, mental health, emergency department, pandemics, psychological distress, psychiatry

## Abstract

**Background:**

Coronavirus disease 2019 (COVID-19) has impacted on a range of physical, mental and societal health indices. Increased levels of psychological stress are often reported following pandemics.

**Aim:**

To describe and compare the presentations of mental health care users pre- and post-initiation of the lockdown, with an emphasis on demographic profiles and final diagnoses.

**Setting:**

The study was conducted as a retrospective record review over the predetermined period at a large public hospital in Johannesburg and included all mental health care users requiring psychiatry consultation during the study period.

**Methods:**

Clinical records were traced via the emergency department registration desk, and information pertaining to demographics, presenting complaints, date of presentation and diagnosis was extracted.

**Results:**

A significant increase was seen in patients with psychotic disorders from pre-COVID-19 to post-COVID-19. There was a reduction in presentations of mood disorders and substance-related disorders. Patients presenting in the post-COVID-19 time period were significantly younger than in the pre-COVID-19 time period.

**Conclusion:**

Pandemics result in notable negative mental health sequelae. Policies aimed at mitigating the spread of infective agents should be implemented with consideration of the burden of psychological distress following the pandemic.

**Contribution:**

This study provides insights into clinical and demographic variables in a mental health care population serviced at a government hospital pre- and post-COVID-19 lockdown regulations.

## Introduction

A novel severe acute respiratory syndrome coronavirus 2 (SARS-CoV-2) was identified as an aetiological agent in cases of pneumonia seen in the Chinese city of Wuhan on 31 December 2019.^1^ The virus subsequently spread across the world, and the coronavirus disease 2019 (COVID-19) pandemic was declared by the World Health Organization on 11 March 2020.

The global COVID-19 pandemic posed unprecedented challenges for societies, economies and healthcare systems.^2^ Individual and government responses to this major public health emergency affected millions of people and changed people’s ways of socialising, working, studying and living.^3^

The COVID-19 outbreak is the greatest global pandemic experienced in over a century, and it has impacted on a range of physical, mental and societal health indices.^4^ Increased levels of psychological stress, including post-traumatic stress, major depression and suicide, are commonly reported after mass disasters and pandemics.^5^ Similar to conditions faced in South Africa, the novel and dramatic societal shifts brought by the national and international social policies aimed at mitigating the spread of COVID-19 are understood to underlie psychiatric presentations and exacerbate the existing clinical conditions.^6^ While there are many different aspects to how an infectious disease outbreak and a natural disaster affect mental health, there are also similarities.^7^ Emergency departments are one of the first points of contact with patients, hence they present a unique opportunity to observe the effects of the COVID-19 pandemic on the mental health of the population.^8^

The National Department of Health developed mental health guidelines during the COVID-19 pandemic with the aim to promote and protect the mental well-being of the citizenry.^9^ The provision of mental health care was permitted during the lockdown period, and virtual and telephonic services were made available for those experiencing psychological distress.

The effect of the pandemic in a study conducted by the Human Sciences Research Council reported that 33% of South Africans were depressed, while 45% were fearful, and 29% were experiencing loneliness during the first lockdown period.^9^ These statistics are likely to be worse following the COVID-19 pandemic as a result of multiple factors that negatively affected people’s mental health. A report by Nguse and Wassenaar suggests that there had been significant suffering by many South Africans during the lockdown period.^9^

Migrants and refugees were particularly vulnerable during the pandemic, both because of potentially precarious housing environments and because of increased stigma and discrimination, in addition to restrictions on their movement and rights.^10^ Under normal conditions, international migrant workers have a high burden of common mental disorders and a lower quality of life than the local population.^11^

In Australia, Kam et al. described changes in patterns of presentations to emergency departments during COVID-19 lockdowns, including higher presentations with mental health problems in 2020 compared to 2019.^12^ In the United Kingdom, lockdown resulted in fewer psychiatric presentations, but those who presented were likely to have severe symptoms.^13^ During the pandemic, individuals were required to balance their need for urgent mental health care against the risk of infection in hospital settings, possibly impacting on help-seeking behaviour.^5^

A systematic review and meta-analysis conducted in the early stages of the COVID-19 pandemic identified female gender, younger age and lower socioeconomic status as factors associated with psychological distress during the pandemic.^14^ Younger age was associated with higher odds of anxiety and depression.^14^ Current employment was associated with lower odds of psychological distress.^14^ Past mental health treatment also emerged as a significant predictor for anxiety and depression associated with the COVID-19 pandemic.^15^

Early impacts of COVID-19 globally demonstrated rates of generalised anxiety disorder ranging between 19.6% and 35.1% and rates of major depressive disorder ranging from 9.8% to 48.3%.^15^ Individuals who recovered from physical SARS-CoV-2 symptoms experienced higher odds of anxiety and depression.^16^

The aim of this study was to describe and compare the presentations of mental health care users before and during lockdown, with an emphasis on the number of presentations, demographic profiles and types of presenting symptoms and subsequent diagnoses given to patients.

The objectives were to describe and compare in the year prior to, and after the initial COVID-19 lockdown:

- The number of mental health-related emergency department presentations to a tertiary hospital in Johannesburg.
- The clinical and demographic variables of mental health care users presenting to the emergency department at Charlotte Maxeke Johannesburg Academic Hospital (CMJAH) during times of high population stress.

## Research methods and design

### Study design and setting

The study was conducted as a retrospective record review over the predetermined period at CMJAH and included all mental health-related patients requiring psychiatry consultation during the study period.

### Sample population

The study population included all adults with new and pre-existing mental health-related diagnoses presenting in the predetermined period. Medical records of adult patients presenting between 01 March 2019 – 29 February 2020 (identified as the pre-COVID-19 lockdown period) and 01 March 2020 – 28 February 2021 (identified as during COVID-19 lockdown period) were reviewed. Medical records of adults with complete demographic information and diagnoses were included, while those of patients under the age of 18 years and those patients with non-mental health-related diagnoses were excluded.

### Data collection

Once the required approvals were obtained, medical records were reviewed by the first author, who is a psychiatry registrar, to collect the data. All files were traced via the emergency department registration desk. Data were collected using a structured data collection sheet, and information pertaining to demographics, presenting complaint or symptoms, duration of hospital stay and diagnosis was extracted. Diagnoses were captured as per the Diagnostic and Statistical Manual of Mental Disorders, 5th Edition (DSM-5). The data were collected and recorded onto a Microsoft Excel data sheet ensuring the removal of all identifying data.

### Statistical data analysis

Statistical analyses were conducted in R software (version 4.00; www.R-project.org). Tests are two-tailed and the model significance was set at 0.05. The results are reported descriptively as counts and percentages for categorical data, and means and standard deviation for continuous data. Welch’s *t*-test was used to analyse the length of hospital stay pre-COVID-19 and during COVID-19 lockdown period. Pearson’s chi-squared analyses was used to determine if there was a correlation between sociodemographic variables and the final diagnosis.

### Ethical considerations

Permission to conduct the study was obtained from the Chief Executive Officer (CEO) of the hospital and ethical clearance to conduct this study was obtained from the University of the Witwatersrand, Human Research Ethics Committee (No. M220384).

## Results

### Sociodemographic profile

A total of 101 files were retrieved for the first time period, of which 18 patients were not referred to psychiatry even though they were registered as having presented with psychiatric symptoms. This was either because of an error in registration or more likely that after assessment by the emergency department team, it was deemed that the presentations were not psychiatric. Three files could not be retrieved from records. For ease of comparison to the second time period, 80 files were then reviewed. There was an overall reduction in the total number of patients seen during the lockdown period compared to the pre-COVID-19 time period, with 550 presenting in the first time period and 474 presenting in the lockdown period. The biggest change was seen in April 2020, which was the first full month after the declaration of stage-5 lockdown, with a 50% reduction compared to the previous year.

**FIGURE 1:**
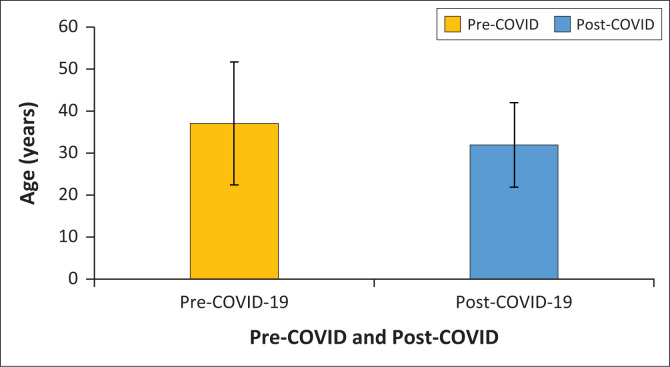
The mean (± SD) ages of patients treated pre-COVID-19 and post-COVID-19 at the Charlotte Maxeke Johannesburg Academic Hospital emergency department.

Patients were significantly older, by an average of 6 years, in the pre-COVID-19 lockdown period compared to the post-COVID-19 lockdown period (*t*-test = 2.61, *p* = 0.010) (Figure 1).

**FIGURE 2:**
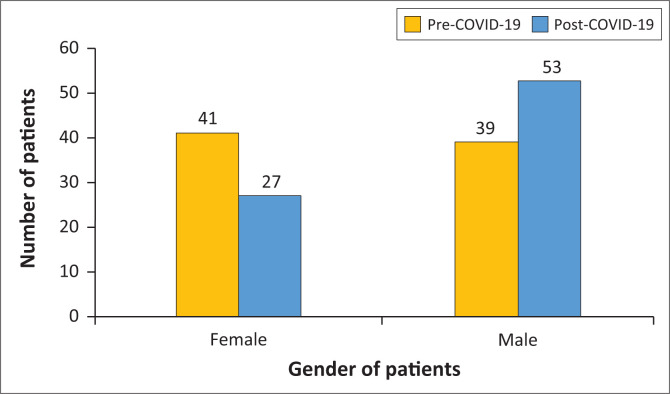
The number of male and female patients treated pre-Covid and post-Covid at the CMJAH emergency department. The numbers of patients are shown above the bars.

Gender was a significant predictor of the number of patients treated pre- and post-COVID-19 (*p* = 0.039). A greater proportion of females were treated pre-COVID-19 (60%) than post-COVID-19 (40%), whereas fewer males (43%) were treated pre-COVID-19 than post-COVID-19 (57%) (Figure 2).

**FIGURE 3:**
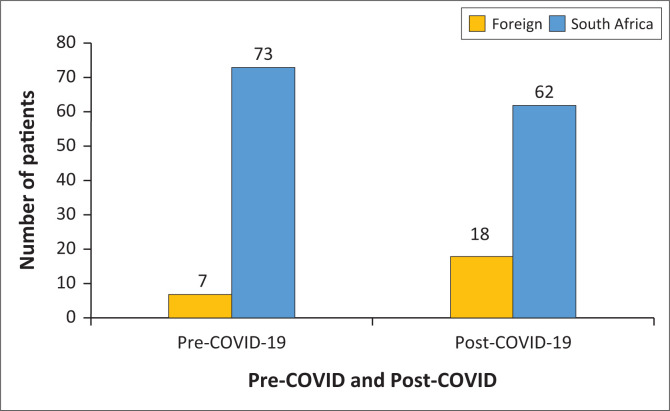
The number of foreign nationals and South African patients treated pre-COVID-19 and post-COVID-19 at the Charlotte Maxeke Johannesburg Academic Hospital emergency department. The numbers of patients are shown above the bars.

Most patients were of South African origin both pre- and post-COVID-19. However, grouping all foreign nationals together in comparison to the proportion of South Africans showed a significant difference (Fisher’s exact test: *p* = 0.030), with the proportion of foreign nationals increasing from 8% to 22% and the proportion of South Africans decreasing from 92% to 78% from pre-COVID-19 to post-COVID-19 (Figure 3). Foreign nationals were three times (odds ratio) more likely to be seen post-COVID-19 than pre-COVID-19.

Most patients were single and expressed high school as their highest level of education. Most patients were also unemployed (Table 1).

**TABLE 1:** Sociodemographic characteristics of patients treated pre- and post-COVID-19 at the Charlotte Maxeke Johannesburg Academic Hospital emergency department.

Socio-demographics	Pre-COVID-19		Post-COVID-19		Statistics
	*n*	%	*n*	%	
**Relationship status**					χ^2^ = 2.61, *df* = 3, *p* = 0.456
Single	69	86	63	79	
Married	5	6	7	9	
Divorced	4	5	4	5	
Widowed	2	3	6	8	
**Highest level of education**					χ^2^ = 4.44, *df* = 2, *p* = 0.108
Primary school	5	6	4	5	
High School	55	69	66	83	
Tertiary	20	25	10	13	
**Employment status**					χ^2^ = 5.83, *df* = 4, *p* = 0.213
Full time	13	16	13	16	
Part time	4	5	2	3	
Pensioner	5	6	1	1	
Unemployed	51	64	61	76	
Other	7	9	3	4	

Values are counts (percent). Statistics = Pearson’s chi-squared tests.

Significantly more patients had a psychotic disorder (*p* = 0.02) presenting during COVID-19 lockdown than the pre-COVID-19 period (Table 2).

**TABLE 2:** Clinical characteristics of patients treated pre- and during Covid at the CMJAH emergency department.

Final diagnoses	Pre-COVID-19		Post-COVID-19		Statistics
	*n*	%	*n*	%	χ^2^ = 13.44, *df* = 6, *p* = 0.037
Anxiety disorder	0	0	1	1	-
Cognitive disorder	2	3	0	0	-
Mood disorder	32	40	22	28	-
Personality disorder	7	9	3	4	-
Psychotic disorder	21	26	39	49	-
Substance-related disorder	16	20	11	14	-
Other	2	3	4	5	-

CMJAH, Charlotte Maxeke Johannesburg Academic Hospital.

## Discussion

### Key findings

Our study showed an overall reduction in the total number of mental health-related presentations to the emergency department (ED). This contrasted with early evidence on psychiatric morbidity from China, which indicated that the rates of mental illness during the COVID-19 pandemic were expected to rise rather than fall.^17^ Although patients with physical complaints may have been deterred from presenting to the ED during the height of the pandemic, patients suffering from significant psychological distress may have been less likely to be deterred.^18^

Patients with anxiety and depression were similarly deterred from presenting to the ED because of COVID-19, yet the nearly steady number of visits for anxiety and depression during the pandemic reflects an increased prevalence of acute psychiatric problems in the general population.^18^ This contrasted with the results found in our study, which showed that the majority of mental health presentations were attributable to psychotic disorders. The acute onset of psychosis or occurrence of relapse is a medical emergency,^19^ and this is a possible explanation for the higher number of psychotic presentations seen in our study. Apart from distress and behavioural dysfunction associated with psychosis, there may be danger to the patient and others.^19^

Pérez-Balaguer et al. suggested that the intense psychosocial stress associated with a new life-threatening disease and national lockdown restrictions could be triggers for new-onset psychotic disorders.^20^ This same study found presentations meeting criteria for acute and transient psychotic disorders, likely triggered by stress generated by COVID-19.^20^ Research conducted in the aftermath of the Spanish influenza of 1918 demonstrated an association between exposure to general respiratory viruses and subsequent psychotic episodes. Pandemics such as the Spanish flu demonstrated that these viruses can have a more immediate effect, resulting in cases of acute psychosis soon after or at the time of infection.^6^ Restrictions on the availability of transport may have also led to decrease follow-up at clinics, nonadherence and subsequent relapses.

Mood disorders, particularly depressive disorders, were the second most prevalent in our study. This could be as a result of a variety of reasons. Movement restriction during lockdown negatively affected many people’s earning potential. Additionally, people spent extended periods of time isolated, with limited opportunities for social interactions. Isolation and confinement, even if only for a few weeks, can cause lasting psychological problems.^21^ Longer quarantine duration and infection fears could serve as stressors leading to serious emotional and psychological distress.^3^ Infection with the COVID-19 virus itself may increase the risk of developing mood disorders. Direct infection of COVID-19 and the collateral impacts of the pandemic place many at risk for a variety of mood disorders.^6^ While the percentage of mood disorders showed a decline in the post-lockdown period, it was significantly higher than that reported in a study by Kim et al.,^22^ which showed that 14.5% of adults displayed significant depressive symptoms.

Our study showed a higher proportion of males presenting in the post-lockdown period, which was in contrast to a study conducted by Di Lorenzo et al.^23^ in Italy, which showed a high prevalence of females in both time periods. The difference in our results could possibly be explained by the unavailability of maladaptive coping mechanisms that males with psychological distress sometimes employ, such as the use of illicit substances,^24^ which were not readily available during lockdown. A study done in Thailand^25^ found higher rates of mood disorders in women than in men. As our study showed a decline in the presentation of mood disorders post-COVID-19, this could also account for the fewer female presentations.

Stroever et al.^26^ found that the most common mental health presentation in New York during the early stages of the pandemic was alcohol abuse with intoxication. This finding was similar to a study by Munich et al.,^27^ who found that substance use disorder-related ED visits tend to increase in early pandemics. These findings were in contrast to our study which showed a relative reduction in substance-related disorders. Premises selling alcohol were closed as part of lockdown regulations,^28^ which likely contributed to this reduction.

The population in our study was significantly younger in the post-lockdown period than in the pre-lockdown period. Severe COVID-19 infection was associated with medical comorbidities, which are more prevalent in older people. This could explain why older people would be more reluctant to present to the ED, where the chance of infection could potentially be higher. Similar to our study, a study conducted in China^24^ found that more young people had a tendency towards psychological problems. This was related to negative coping strategies and low education level.^24^

There was a significant increase in the proportion of foreign nationals treated in the post-lockdown period than there was in the pre-lockdown period, which was similar to other studies. Ambrosetti et al.^29^ in Switzerland found that there was a significant increase in the number of migrants being admitted to psychiatric EDs in the post-lockdown period. Infection prevention measures associated with the lockdown may have impacted the lives of many migrants, as they faced increased precariousness, financial constraints and stigmatisation by the non-migrant community.^29^ Vigo et al. found that migrants and refugees were particularly vulnerable during the pandemic both because of potentially precarious housing environments and because of increased stigma, discrimination, and restrictions on their movements and rights.^10^

### Strengths and limitations

Our study was conducted as a retrospective chart review, which has some limitations, as well as recognised advantages. Scientific and systematic investigation of existing health records is an important and valued methodology in healthcare research. Retrospective chart review is an important methodology with distinct advantages and has the potential to provide us with valuable research opportunities.^30^ There is limited comparative research in the same institution reviewing the impact of the lockdown period on the mental health population. This study aimed at describing the demographic and clinical profiles that may have been impacted by the regulations of a lockdown protocol. Our data include information from 1 month where the lockdown was not in effect for the full calendar month, which may have minimally skewed the results. Albeit a limited sample size for each time period, generalisation of our results may be made for similar population groups. Although our study was conducted at a single centre, CMJAH serves a diverse population, which is representative of most urban areas in South Africa. The findings of the study highlight the importance of prioritising preventative strategies for mental health for young and healthy individuals, male patients and foreign patients and stresses the emphasis of clinical patterns in our setting that differ from that of international studies. Further information may derive in anticipating vulnerable populations in a pandemic which may direct care, protocol development and policy guidance.

## Conclusion

Our study highlighted the negative effect that pandemics can have on mental health populations. The implementation of policies aimed at curbing the spread of the pandemic needs to be a careful balancing act between protecting people from infection by the causative agent and protecting their mental health. Many factors contribute to good mental health, including social relationships and financial security, and these seem to be most disrupted during pandemics. Vulnerable populations such as the young, the elderly and those who have immigrated from other countries appear to be more negatively affected, necessitating pointed strategies designed to reduce this disparity.
